# Current Trends in Irrigation Solution and Adjunct Use During Endodontic Therapy Among Dental Professionals in Jeddah, Saudi Arabia: A Cross-Sectional Study

**DOI:** 10.7759/cureus.32168

**Published:** 2022-12-03

**Authors:** Ziyad T Alzamzami, Assalah A Alqurashi, Lolo A Almansour, Heba M Ashi, Ayman M Abulhamael, Faisal T Alghamdi, Maysoon T Albahiti

**Affiliations:** 1 Endodontics, Faculty of Dentistry, King Abdulaziz University, Jeddah, SAU; 2 Dental Services, King Fahad Armed Forces Hospital, Jeddah, SAU; 3 Advanced General Dentistry, The University Dental Hospital, King Abdulaziz University, Jeddah, SAU; 4 Dental Public Health, Faculty of Dentistry, King Abdulaziz University, Jeddah, SAU; 5 Oral Biology, Faculty of Dentistry, King Abdulaziz University, Jeddah, SAU

**Keywords:** cross-sectional survey, irrigation adjuncts, irrigation, sodium hypochlorite, endodontic therapy

## Abstract

Introduction

Chemical irrigation is a crucial component of endodontic therapy, and irrigation adjuncts increase the efficiency of non-surgical root canal system disinfection, reduce microbial loads, and enhance the penetration of irrigants throughout the root canal system. This study aimed to determine the current trends in chemical irrigation and its adjunct use during root canal therapy by general dental practitioners (GDPs) and endodontic specialists in both government and private sectors in the city of Jeddah, Saudi Arabia.

Methods

This cross-sectional study used a self-administered survey sent to GDPs and endodontic specialists in both government and private sectors in the city of Jeddah, Saudi Arabia. Responses were accepted from November 2019 till May 2020. The survey was randomly distributed to consenting participants. The results are presented using descriptive statistics.

Results

A total of 302 participants responded to the survey, with a 44% response rate. The majority of responses were from GDPs (54%), while 46% were from endodontic specialists. Regarding the workplace, 59% of respondents were public sector professionals, 25% were private sector professionals, and 16% were both public and private sector professionals. Around 30% of the respondents used sodium hypochlorite (NaOCI) as their primary irrigation solution during root canal treatment, with 52% using it at its full-strength concentration and 18% and 17% preferring to use it in combination with Ethylenediaminetetraacetic acid (EDTA) only or EDTA and saline, respectively. When asked to rank the reasons for choosing their irrigant of choice during therapy; antibacterial capability and tissue dissolution were the most crucial factors to 80% and 57% of participants, respectively. Half of the respondents irrigated the canal to 2 mm from the apex and 21% irrigated to 1 mm from the apex. Three-quarters of participants aimed to remove the smear layer during root canal treatment. Only 47% of respondents used adjuncts to irrigation, and 71% reported that their choice of irrigation solution would differ depending on whether the apex is open or closed.

Conclusion

Most respondents used full-strength NaOCI concentration as the main irrigation solution and routinely removed the smear layer during root canal treatment. Only 47% of respondents used irrigation adjuncts such as ultrasonics.

## Introduction

Irrigation plays a significant role in endodontic therapy; successful treatment is predicated on achieving thorough chemo-mechanical cleansing of the root canal system to maintain periapical health and integrity. Thus, microorganisms that either remain in the root canal system after disinfection or recolonize and survive in the filled spaces are the main causes of endodontic treatment failure [1]. Zehnder proposed a set of ideal properties of a chemical solution for root canal irrigation [2]: it must be non-antigenic, non-toxic, non-carcinogenic, and non-caustic to periodontal tissues; have a low propensity to cause anaphylactic shock; have a broad-spectrum antimicrobial effect (substantivity); and it must remove the smear layer and be able to dissolve vital and necrotic pulp tissue and inactivate endotoxins [2, 3]. Other desirable properties of an irrigant include being inexpensive, easy to use, does not cause tooth discoloration, and promotes the sealing of filling materials [3, 4]. No available irrigant can yet be regarded as completely “ideal” [5, 6], and usually, two or more chemical solutions are required in combination for effective irrigation and complete removal of debris and the smear layer [3, 7].

The most popular irrigant used in treating non-surgical root canals is sodium hypochlorite (NaOCl) [7-9] at a concentration of 0.5 to 6% [7, 10]. There is little proven difference in efficacy between concentrations [8, 11], and there is no difference between 0.5 and 6% with respect to tissue dissolution [12]. Since it meets most of the criteria of an ideal irrigant despite its unpleasant smell and taste, toxicity, and limited removal of inorganic tissue, NaOCI has become the recommended irrigant of choice [13].

The 2% of chlorhexidine (CHX) solution is also a popular irrigant and intracanal medicament in endodontic therapy [1, 7] due to its prolonged antimicrobial effect (substantivity) [3]. However, CHX does not dissolve tissue, so it cannot fully replace NaOCI as a sole irrigant [1, 3, 7]. Complete disinfection of the root canal system necessitates the use of an irrigant that can dissolve inorganic tissue. Ethylenediaminetetraacetic acid (EDTA) and citric acid (CA) have been investigated as chelating agents to effectively remove the smear layer [7], but these chemicals have weak or no antibacterial effect. Moreover, Torabinejad et al. recently introduced a mixture of tetracycline isomer, acid, and detergent (MTAD), which was shown to eradicate the smear layer and possess antimicrobial activity [3, 14]. While MTAD can be used instead of EDTA [7], it should be regarded as an adjunct to NaOCI rather than a replacement [11]. Other irrigants include sterile water and saline, which have no tissue-dissolving capability nor antimicrobial activity so should not be used as the main irrigant but instead to wash out between chemical solutions to prevent harmful byproducts [7].

The internal anatomy of the root canal system can be complicated by the presence of isthmuses, accessory and lateral canals, curvatures, and other irregularities that challenge the effective elimination of microbes and debris through mechanical means alone. Hence, chemical disinfection with the use of irrigation adjuncts is extremely valuable in such cases. To enhance the effectiveness and penetration of chemical solutions inside the root canal system, several irrigation adjuncts have been proposed, such as agitation with a syringe and needle or gutta percha points with an in-and-out pumping motion [11]. Systems such as Endo Activator, a sonic-driven device, work on the principle of hydrodynamic agitation [11, 15], while EndoVac can safely deliver the irrigant to the apices and remove debris effectively by negative pressure [11].

In a study investigating the awareness of decontamination solutions and techniques during root canal therapy among dentists in Jeddah, Saudi Arabia, the author found that NaOCI was the most commonly used irrigant while only 14.3% of practitioners used chelating agents [16]. To identify the requirement of intervention and remediation to improve the overall success of root canal therapy, dentists must be aware of current trends and expand their knowledge and understanding of chemical irrigant and their applications. Thus, we aimed to determine current trends in the use of all the available chemical irrigants and adjuncts to irrigation during root canal therapy by general dental practitioners (GDPs) and endodontic specialists in both government and private sectors in the city of Jeddah, Saudi Arabia.

## Materials and methods

Setting

The Research Ethics Committee of the Faculty of Dentistry at King Abdulaziz University, Jeddah, Saudi Arabia approved the study protocol (#352-12-21), and the Declaration of Helsinki for the ethical conduct of research on participants was followed throughout. This questionnaire-based survey was distributed electronically among 685 participants. Government sector dentists and endodontic specialists were recruited from all four major dental hospitals in Jeddah (King Fahad General Hospital, King Abdulaziz University Dental Hospital, East Jeddah General Hospital, and King Fahad Armed Forces Hospital), while others were randomly selected from primary healthcare centers across Jeddah’s five major districts (North, South, Middle, East, and West). Similarly, private sector dentists and endodontic specialists were randomly selected from private hospitals and polyclinics in the same five major districts. Data were collected between November 2019 and May 2020 with open- and closed-ended questions. A validated questionnaire used in a previous study [17] was modified into English to accommodate the objectives of the present study.

Sample size calculation

The sample size was calculated using an online calculator (www.raosoft.com) (Raosoft, Inc., Seattle, WA, USA). The sample size calculation was based on a population size of 685 and 95% confidence intervals with a margin of error of 5%, which yielded a sample size of 247 participants (Appendix 1).

Questionnaire

The modified questionnaire comprised three main domains and a total of 16 open- and close-ended questions consisting of numerical rankings, multiple choice questions, and multiple selections with options for free text answers that were appropriate (Appendix 2). The first domain gathered demographic information, including practice setting and rank of specialty; the second domain was on chemical irrigant utilization, and the third domain focused on irrigation adjunct utilization. The latter two domain questions were designed to obtain all relevant information regarding irrigation including irrigant selection, irrigant concentration, the reason for irrigant selection, smear layer removal, the gauge of the needle, depth of needle penetration, duration of irrigation, the volume of irrigating solution used, the use and type of irrigant agitation device, and the use of adjuncts to irrigation in different endodontic treatments.

Administration of the questionnaire

This was a descriptive cross-sectional study of GDPs and endodontic specialists conducted through a self-administered, structured questionnaire. The modified questionnaire was pre-tested on ten dental professionals (five GDPs and five endodontic specialists) from various dental hospitals in Jeddah who were not part of the study sample. The results of pre-testing the questionnaire were to make the cross-sectional survey more reliable and valid after modifications were made. These questions provided new answer options to determine all the available irrigants used during endodontic treatment in their practices. The inclusion criteria included Saudi dentists who were GDPs and endodontic specialists who had master’s degrees or advanced clinical certificates in endodontics only. Privacy was guaranteed, and the participant outcomes remained confidential in this cross-sectional survey.

Statistical analysis

Data was coded and entered into a database system for analysis using Statistical Package for the Social Sciences (SPSS) version 26.0 for Windows (IBM Corp., Chicago, IL, USA). The analysis included simple descriptive statistics in the forms of percentages and frequency distributions visualized using pie and bar charts.

## Results

**Figure 1:**
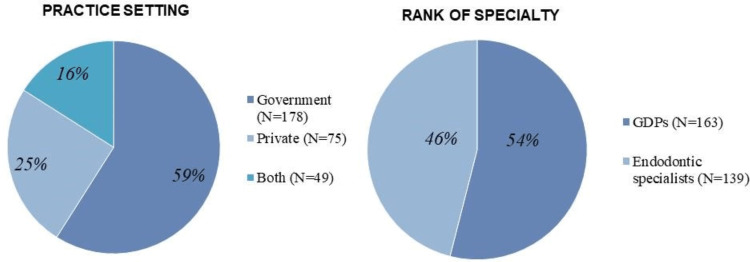
The frequency distributions and percentages of practice settings and rank of specialty.

Of the 685 self-reported questionnaires distributed to GDPs and endodontic specialists with one to 30 years of clinical experience in government and private dental sectors in Jeddah, 302 self-reported questionnaires were completed (44% response rate). The majority of responses were from GDPs 163 (54%), while 139 (46%) were from endodontic specialists. One-hundred and seventy-eight (59%) respondents were public sector workers (Figure 1). 

**Figure 2:**
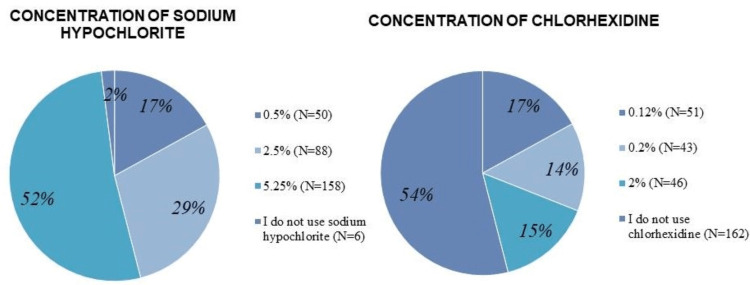
The frequency distributions and percentages of concentrations of sodium hypochlorite and chlorhexidine used.

**Figure 3:**
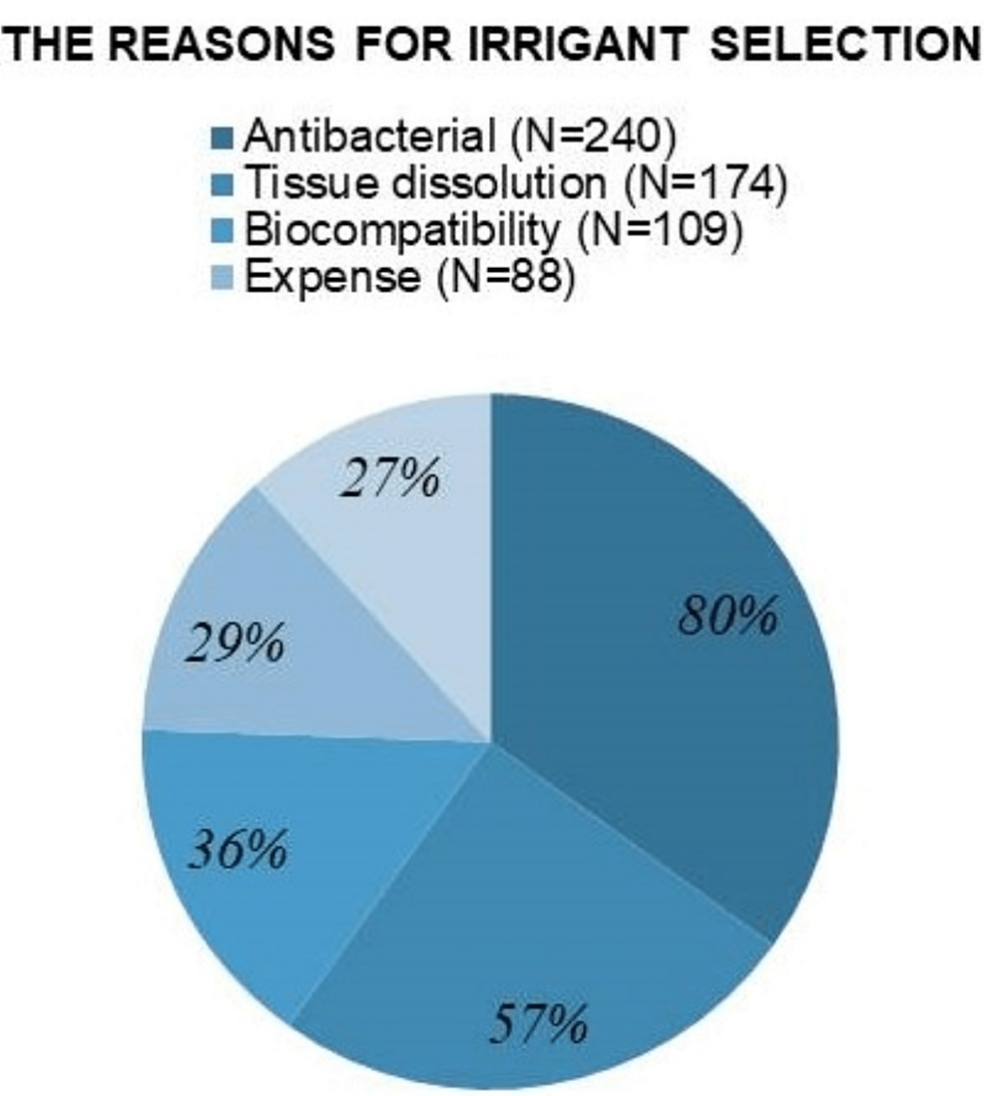
Responses to questions regarding the reasons for primary irrigant selection.

Eighty-nine (30%) respondents primarily used NaOCI as the main chemical irrigation solution alone, while 54 (18%) and 52 (17%) used it in combination with other solutions including EDTA only or EDTA and saline, respectively (Table 1). A slight majority of respondents 158 (52%) indicated that they used full-strength NaOCI (concentration ≥5.25%). With respect to chlorhexidine usage, 54% of respondents (n=162) indicated that they did not use CHX in their endodontic treatment (Figure 2). Participants were asked to rank the reasons for their choice of the primary irrigation solution they use; antibacterial capability was reported to be the most important reason (n=240; 80%) followed by tissue dissolution ability (n=174; 58%), biocompatibility (n=109; 36%), cost (n=88; 29%), and substantivity (n=83; 27%) (Figure 3).

**Table 1:** The frequency distributions and percentages of chemical solutions used as the primarily irrigant. EDTA: *Ethylenediaminetetraacetic acid, *MTAD: *mixture of tetracycline isomer, acid, and detergent*.

Type of chemical solution	Frequency (%)
Sodium hypochlorite	89 (29.5%)
Saline	6 (2. 0%)
Sodium hypochlorite; EDTA	54 (17.9%)
Sodium hypochlorite; saline	43 (14.2%)
Sodium hypochlorite; chlorhexidine	3 (1. 0%)
Sodium hypochlorite; chlorhexidine; EDTA	16 (5. 3%)
Sodium hypochlorite; saline; EDTA	52 (17.2%)
Sodium hypochlorite; chlorhexidine; saline	13 (4. 3%)
Sodium hypochlorite; sterile water; EDTA	2 (0.7%)
Sodium hypochlorite; chlorhexidine; saline; EDTA	14 (4. 6%)
Sodium hypochlorite; chlorhexidine; EDTA; other	1 (0.3%)
Sodium hypochlorite; chlorhexidine; EDTA; MTAD	2 (0.7%)
Sodium hypochlorite; chlorhexidine; saline; EDTA; MTAD	2 (0.7%)
Sodium hypochlorite; saline; EDTA; citric acid	1 (0.3%)
Sodium hypochlorite; chlorhexidine; saline; sterile water; EDTA; MTAD	1 (0.3%)
Sodium hypochlorite; chlorhexidine; saline; sterile water; EDTA	1 (0.3%)
EDTA	1 (0.3%)
Sterile water	1 (0.3%)
Total	302 (100.0%)

**Figure 4:**
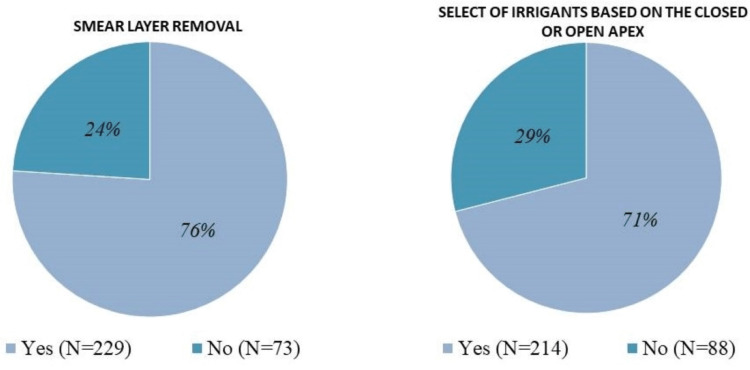
Responses to questions regarding the smear layer removal and selection of irrigants based on a closed or open apex.

Two hundred and twenty-nine (76%) respondents aimed to remove the smear layer during endodontic therapy (Figure 4), and 214 (71%) respondents reported that their selection of irrigant would change depending on whether the apex is open or closed (Figure 4).

One hundred and eight (36%) respondents used 10 mL of irrigating solution, with half (n=151, 50%) irrigating the canals to 2 mm and 62 (21%) to 1 mm from the apex, with 27- and 30-gauge needles preferred in 135 (45%) and 108 (36%) of respondents, respectively (Table 2). Ninety-five (32%) respondents applied irrigant for 30 seconds to 1 minute per canal, while 64 (21%) respondents applied irrigant for over 2 minutes per canal (Table 2).

**Table 2:** Responses to questions regarding the syringe irrigation and irrigation technique. mm: *Millimeter, *mL: *Milliliter*

Question	Responses	Frequency (%)
Routine gauge of the needle	26 gauge	31 (10.3%)
	27 gauge	135 (44.7%)
	30 gauge	108 (35.8%)
	31 gauge	28 (9. 3%)
Depth of penetration of needle	1 mm from apical foramen	62 (20.5%)
	2 mm from apical foramen	151 (50.0%)
	3 mm from apical foramen	51 (16.9%)
	4 mm from apical foramen	38 (12.6%)
Duration of irrigation per canal	<30 seconds	90 (29.8%)
	30 seconds - 1 minute	95 (31.5%)
	1 - 2 minutes	53 (17.5%)
	>2 minutes	64 (21.2%)
Volume of irrigating solution	5 mL	73 (24.2%)
	10 mL	108 (35.8%)
	15 mL	61 (20.2%)
	>15 mL	60 (19.9%)
Total		302 (100.0%)

**Figure 5:**
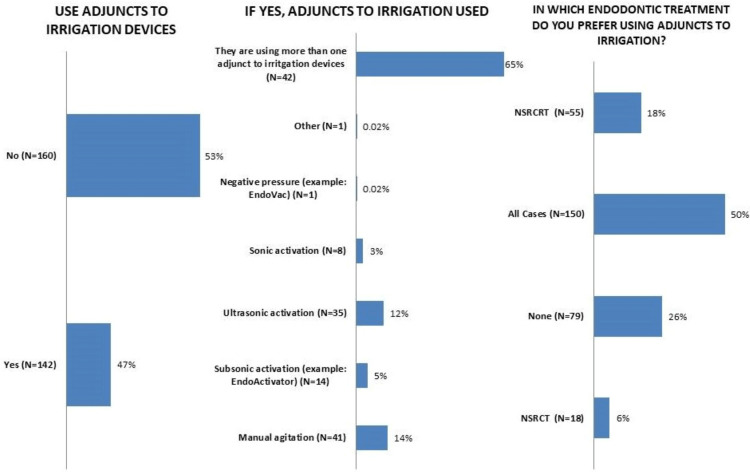
Responses to questions regarding the irrigation adjunct utilization. NSRCRT: *non surgical root canal retreatment, *NSRCT: *non surgical root canal treatment*.

One hundred and forty-two (47%) respondents used an adjunct to irrigation, of whom 41 (14%) agitated the irrigating solution manually and 35 (12%) used ultrasonics to activate the solution. In addition, only 150 (50%) respondents were using an adjunct to irrigation in all cases including non-surgical root canal treatment and re-treatment (Figure 5).

## Discussion

This cross-sectional survey was adapted from Dunter et al. [17] and modified to accommodate the current study objectives. The aim was to collect data from endodontic specialists and GDPs in the city of Jeddah, Saudi Arabia to determine current trends in the use of chemical irrigants and adjuncts during endodontic therapy. The response rate was 44%, which compares favorably to other similar surveys with a lower response rate of 33.2% (India) [1] and 28.5% (America) [17].

NaOCI is considered the gold standard irrigant for endodontic therapy since it meets most of the criteria defining the “ideal” irrigant solution [8, 18, 19]. On the other hand, CHX is a cationic bis-guanide having antibacterial properties [20]. The combination of NaOCl and CHX solution to strengthen their antibacterial activity has been of great interest, especially due to the substantivity property of CHX which prolongs its local activity [21]. However, the interaction of CHX and NaOCl may produce a chemical form of the brownish orange carcinogenic precipitate “Parachloranaline” that covers dentinal tubules and so interferes with the sealing properties. Moreover, this precipitate is cytotoxic to human cells and can cause tooth discoloration, and therefore the combination of both irrigants is contraindicated [22, 23]. In our cross-sectional survey, participants ranked antibacterial effect and tissue dissolution ability as the two most important qualities affecting selection of the primary irrigation, like the findings from Gopikrishna et al. [1], Dunter et al. [17], and Koppolu et al. [24].

A similar study in 2020 investigated the practices and awareness of decontamination solutions and techniques during root canal therapy among dentists in Jeddah, Saudi Arabia. The author found that NaOCI was the most commonly used irrigant while only 14.3% of practitioners used chelating agents as an adjunct [16]. In contrast, our findings showed nearly one-third of participants (30%) reported the use of NaOCI alone, while 54 (18%) and 52 (17%) used chelating agents as an adjunct in combination with other solutions including EDTA only or EDTA and saline, respectively (Table 1). In addition, only three (1%) of participants combined NaOCI with CHX only. On the other hand, there are various percentages and frequencies concerning the mixture of different chelating agents combined with NaOCI and CHX solutions together or separately as shown in Table 1. The possible reason that led to that contradiction was mainly due to the sample size between both studies (302) participants in our study vs (103) participants in the 2020 study [16].

Using the correct sequence of chemical solutions during irrigation is likely to contribute to a successful outcome of root canal treatments by eliminating the smear layer, reducing bacterial load, and ensuring complete disinfection of the root canal system [8]. There is some controversy regarding the need to remove the smear layer during root canal treatment. The smear layer can become infected and may protect existing bacteria in the dentinal tubules, so it is recommended to remove it, to decrease the bacterial load, permit the flow of irrigating solution into the dentinal tubule, and improve the adaption of the sealers and obturation materials [4, 24]. It has been reported that the antimicrobial effect of irrigating solutions is improved by the complete removal of the smear layer using chelating agents such as EDTA or CA [18], which can easily be achieved with the correct protocols [7]. The recommended time for smear layer removal could range from two to five minutes, especially with thicker layers [2, 25]. We found that the majority of respondents (76%) aimed to remove the smear layer during endodontic therapy (Figure 4), a similar survey for the members of the American Association of Endodontists in 2012, in which 77% of endodontists reported removing the smear layer prior to obturation [17].

Several factors other than the choice of irrigant also play a significant role in effective root canal system disinfection. For example, a higher concentration of NaOCl allows for rapid tissue dissolution [26], and larger volumes and regular irrigant exchange can improve antibacterial efficacy [8]. The preferred concentration of NaOCI was investigated in a subpopulation of dentists in Riyadh, Saudi Arabia. The author found that NaOCl concentration of 2.5%-5% was the most commonly used; selected by 137 (52.7%) of the participants, followed by 54 (20.8%) who used NaOCI concentration of 1%-<2.5%, 30 (11.5%) of participants were using >5%, 13 (5%) were using <1%, and 26 (10%) didn't know the concentration [13]. Our findings illustrated that half of the participants 158 (52%) were using it at full-strength concentration (5.25%), followed by 2.5% of NaOCI concentration among 88 (29%) of participants, 50 (17%) of participants used 0.5% of NaOCI concentration, and 6 (2%) were not using NaOCI at all (Figure 2). There is no consensus on the most suitable concentration of NaOCI to use for irrigation. According to the findings of a previous in-vitro study, the most successful irrigation regimen is 5.25% NaOCl at 40 minutes, whereas irrigation with 1.3% and 2.5% NaOCl at the same time interval is ineffective in eliminating Enterococcus faecalis (E. faecalis) from contaminated dentin cylinders [27]. Based on the results of this study, the author suggested using various irrigants to improve the antibacterial effects during root canal cleaning and shaping [27].

On the other hand, while the optimal time of canal irrigation remains uncertain [2], the longer the irrigant is in contact with the root surfaces, the greater the probability that microbes will be effectively killed, and the bacterial load reduced [11, 28, 29]. The chlorine of hypochlorite rapidly depletes and may no longer be effective after two minutes, but this can be overcome during treatment by using regular irrigant exchange [11, 30]. In our findings, the vast majority of the participants believed that the optimal time of canal irrigation should be between 30 seconds and 1 minute by 95 (31.5%) and followed by less than 30 seconds in 90 (29.8%) participants (Table 2).

Guerreiro-Tanomaru et al. reported that 30-gauge needles with side and apical openings result in better apical disinfection [31]. In our cross-sectional survey, the majority of respondents used 27- and 30-gauge needles to deliver irrigant solutions (Table 2), in line with Haapasalo et al., who reported that small size 27-gauge or preferably 30-gauge needles should be used to gain access to the apical canal [7].

Stojicic et al. showed that agitation with NaOCI enhanced canal cleaning 12-folds compared with irrigation without agitation [26], consistent with previous studies showing that using irrigation devices cleans the canal better than conventional needle irrigation [32, 33]. Dutner and colleagues [17] reported that almost half of their respondents used an adjunct to irrigation, like the 47% reported in this study (Figure 5). The majority of participants who used adjuncts to irrigation activated the chemical solution manually (14%) followed by ultrasonics (12%) (Figure 5). Manual activation may be favored over ultrasonics because it is a low-cost technique that does not require special devices. This finding is similar to that obtained by Monardes et al. [34], who reported that most of their respondents used manual activation.

Our study has some limitations. The data depended on self-reported questionnaires, which may over- or underestimate answers and introduce other biases. This cross-sectional study was conducted as a preliminary study to evaluate the chemical agents in one city in proximity to the main and primary dental hospitals, centers, and clinics. The general practitioners and endodontic specialists who live and practice in Jeddah city are representative of the whole Saudi dental professionals community in one region (Makkah Region) one of the regions of the Kingdom of Saudi Arabia. More studies are needed in other cities of the same region and different regions of the Kingdom of Saudi Arabia to collect more knowledge about the current trends in irrigation protocols. Some of the factors measured in our cross-sectional survey may have been subject to recall bias, such as the duration of use of irrigation and the volume of irrigating solution used per canal. Clinical studies now need to be undertaken to evaluate different irrigation protocols and associate them with root canal treatment outcomes.

## Conclusions

Overall, the results of the present study were encouraging, although dentists should be encouraged to update their knowledge regarding optimal chemical solution utilization and the importance of activation either manually or with specific devices. Most responders used full strength NaOCI as their irrigation solution of choice and routinely aimed to remove the smear layer. Half of them use adjuncts to irrigation, mainly manual and ultrasonic activation. The antibacterial capability and tissue dissolution ability were the two most crucial factors dictating the choice of irrigating solution used, which are the most important properties of NaOCI.

## Appendices

Appendix 1

Sample Size Calculator

^From http://www.raosoft.com/samplesize.html ^

What Margin of Error Can You Accept?

5%

^Note: The margin of error is the amount of error that you can tolerate. If 90% of respondents answer yes, while 10% answer no, you may be able to tolerate a larger amount of error than if the respondents are split 50-50 or 45-55.^

What Confidence Level Do You Need?

95%

^Note: The confidence level is the amount of uncertainty you can tolerate. Suppose that you have 20 yes-no questions in your survey. With a confidence level of 95%, you would expect that for one of the questions (1 in 20), the percentage of people who answer yes would be more than the margin of error away from the true answer. The true answer is the percentage you would get if you exhaustively interviewed everyone.^

What is the Population Size?

685

_Note: How many people are there to choose your random sample from? The sample size doesn't change much for populations larger than 20,000._

*What is the Response Distribution?*

 50%

_Note: For each question, what do you expect the results will be? If the sample is skewed highly one way or the other, the population probably is, too. If you don't know, use 50%, which gives the largest sample size. See below under More information if this is confusing._

*Your Recommended Sample Size is*

247

_Note: This is the minimum recommended size of your survey. If you create a sample of this many people and get responses from everyone, you're more likely to get a correct answer than you would from a large sample where only a small percentage of the sample responds to your survey._

*Alternate Scenarios*

_*With a sample size of 100; 200; 300*: Your margin of error would be 9.06%; 5.84%; and 4.24%, respectively. _

_*With a confidence level of 90%; 95%; and 99%*: Your sample size would need to be 195; 247; and 338, respectively. _

Appendix 2

Survey

Dear Doctor,

You are invited to participate in this research study by completing the attached survey. The following questionnaire will require approximately 5-10 minutes to complete. The purpose of the study is Determine the current trends of chemical solutions irrigation/ adjuncts to irrigation utilization during root canal therapy followed by Endodontists/ Endodontic residents who are members of the Saudi Endodontic Society. Participation is strictly voluntary. Your answers are anonymous, do not put your name on the survey. All answer will be kept confidential. Only group results will be presented, not individual answers. Completion and return of the questionnaire will indicate your willingness to participate in this study.

If you have questions or concerns, please contact us.

Thank you,

Signature: _________________.
 

Demographic Domain:

1.     Practice setting:

a.     Government

b.     Private

c.     Both

2.     What is your rank in Endodontic speciality?

a.     General dentist

b.     Endodontic Specialist

3.     For how many years/months have you been practicing Endodontics?

      ...............  years

     ..............  months

Chemical Solution Irrigation Utilization Domain:

4.     Which irrigants do you primarily use? (Please select all that apply)

a.     Sodium hypochlorite

b.     Chlorhexidine

c.     Saline

d.     Sterile water

e.     EDTA

f.      MTAD

g.     Citric acid

h.     Other   ........................          

5.     Which concentration of sodium hypochlorite do you use?

a. 0.5%

b. 2.5%

c. 5.25%

d. I do not use sodium hypochlorite.

6.     Which concentration of chlorhexidine do you primarily use? a. 0.12%

a. 0.2%

b.   2%

c.  I do not use chlorhexidine.

7.     Rank the reasons for your primary irrigant selection from 1 ( the most important) to 5 (the least important):

a.     Antibacterial capability   ........................                  

b.     Biocompatibility    ........................                 

c.     Tissue dissolution    ........................                

d.     Substantivity    ........................                

e.     Expense       ........................              

8.     Do you routinely aim to remove the smear layer?

a.     Yes

b.     No

9.     Does your choice of irrigant(s) differ based on the closed or open apex?

a.     Yes

b.     No

10.  What is the routine gauge of the needle employed by you during syringe irrigation?

a.     26 gauge

b.     27 gauge

c.     30 gauge

d.     31 gauge

11.  How much depth of penetration of needle do you prefer for irrigation?

a.      1 mm from apical foramen

b.      2 mm from apical foramen

c.      3 mm from apical foramen

d.      4 mm from apical foramen

12.  What is the duration of irrigation do you prefer per canal?

a.      <30 seconds

b.      30 seconds-1 minute

c.      1-2 minutes

d.      >2 minutes

13.  How much volume of irrigating solution do you use?

a.      5 mL

b.      10 mL

c.      15 mL

d.      >15 mL

Irrigation Adjuncts Utilization Domain:

14.  Do you use adjuncts to irrigation/ irrigant agitation devices?

a.     yes

b.     No

15.  If yes, Which, if any, adjuncts to irrigation do you utilize? (Please select all that apply)

a.     Ultrasonic activation

b.     Sonic activation

c. Subsonic activation (example: EndoActivator)

d.     Negative pressure (example: EndoVac)

e.     Manual agitation

f.      Other     ........................                 

16.  In which Endocontic treatment do you prefer using Adjuncts to irrigation?

a.     Non surgical root canal treatment

b.     Non surgical root canal re-treatment

c.     All cases

d.     None
